# Predicting stress levels using physiological data: Real-time stress prediction models utilizing wearable devices

**DOI:** 10.3934/Neuroscience.2024006

**Published:** 2024-04-19

**Authors:** Evgenia Lazarou, Themis P. Exarchos

**Affiliations:** Bioinformatics and Human Electrophysiology Laboratory, Dept of Informatics, Ionian University, GR49132, Corfu, Greece

**Keywords:** stress detection, health monitoring, physical health, mental health, wearables, physiological data, wearable devices, sensor review

## Abstract

Stress has emerged as a prominent and multifaceted health concern in contemporary society, manifesting detrimental effects on individuals' physical and mental health and well-being. The ability to accurately predict stress levels in real time holds significant promise for facilitating timely interventions and personalized stress management strategies. The increasing incidence of stress-related physical and mental health issues highlights the importance of thoroughly understanding stress prediction mechanisms. Given that stress is a contributing factor to a wide array of mental and physical health problems, objectively assessing stress is crucial for behavioral and physiological studies. While numerous studies have assessed stress levels in controlled environments, the objective evaluation of stress in everyday settings still needs to be explored, primarily due to contextual factors and limitations in self-report adherence. This short review explored the emerging field of real-time stress prediction, focusing on utilizing physiological data collected by wearable devices. Stress was examined from a comprehensive standpoint, acknowledging its effects on both physical and mental well-being. The review synthesized existing research on the development and application of stress prediction models, underscoring advancements, challenges, and future directions in this rapidly evolving domain. Emphasis was placed on examining and critically evaluating the existing research and literature on stress prediction, physiological data analysis, and wearable devices for stress monitoring. The synthesis of findings aimed to contribute to a better understanding of the potential of wearable technology in objectively assessing and predicting stress levels in real time, thereby informing the design of effective interventions and personalized stress management approaches.

## 1. Introduction

Stress has become a prevalent concern in modern society, significantly affecting individuals' health and well-being. Its impact can manifest both directly, through physiological effects, and indirectly, through unhealthy behaviors such as excessive alcohol consumption, malnutrition, or poor sleep habits [1]. Therefore, motivating individuals to adapt their behaviors and lifestyles is crucial, while implementing effective strategies to develop stress-prevention mechanisms. This proactive approach becomes critical in preventing elevated stress levels from escalating into more severe health conditions.

Accordingly, the damaging effects of stress extend beyond individual experiences, influencing broader societal dynamics. Recognizing stress' critical role in health outcomes, there is a growing need for innovative approaches to predict and manage stress levels. With the widespread adoption of wearable devices and interconnected devices capable of acquiring high-quality physiological data, there is a growing interest in leveraging these technologies to predict stress levels in real time. Recent literature [2] affirms the feasibility of objectively detecting stress through the analysis of biological data.

This scientific review aims to outline the current landscape of stress assessment, providing insights into the advancements, challenges, and future directions within this rapidly evolving domain. Through the synthesis of existing research, the main objective is to contribute to a nuanced understanding of the potential of wearable technology in objectively assessing and predicting stress levels in real time. With rising concerns regarding stress, this review explores the potential impact of wearable devices on individual and public health outcomes [1]–[3]. It is imperative to underscore the significance of cardiometabolic diseases as a paramount public health concern due to their escalating prevalence worldwide. These innovative technologies facilitate assessing diverse health-related outcomes spanning molecular, clinical, and lifestyle domains. Currently, wearable devices equipped for continuous and longitudinal health monitoring outside traditional clinical settings offer valuable insights into varied populations' health and metabolic status, ranging from healthy individuals to those at different disease stages. We present an overview of the most relevant wearable and digital devices for evaluating indicators of cardiometabolic diseases. Additionally, we discuss how data acquired from such devices can advance our understanding of metabolic disorders, improve diagnostic accuracy, identify early-disease markers, and inform personalized treatment and prevention strategies [4].

Furthermore, the objective is to provide a comprehensive overview of the current knowledge landscape, evaluate the strengths and limitations of previous models and methodologies, and identify research gaps that have yet to be addressed. More specifically, the primary focus areas to be covered include:

i) Physiological indicators of stress: Reference to the physiological changes associated with stress, explicitly emphasizing metrics that wearable devices can record. Wearable sensors can continuously monitor physiological parameters like heart rate (HR) and breathing patterns. This real-time data can offer valuable insights into an individual's mental health. Specifically, variations in HR may serve as an indicator of stress or anxiety [5],[6]. This review will emphasize the current comprehension of the connections between these indicators and stress levels, evaluating the uniformity of these associations across various populations.

ii) Wearable devices in stress monitoring: Reference the current utilization of wearable devices for collecting physiological data in stress prediction. Wearable sensors provide individuals with real-time feedback on indicators or biomarkers of their health, allowing them to recognize patterns that may indicate the need for behavioral interventions [7]. For instance, a wearable sensor can promptly notify an individual of an elevated HR, potentially indicating the presence of stress or anxiety. The review will assess the effectiveness of these devices in real-time monitoring of physiological indicators, evaluating their reliability in measuring stress-related changes. Nevertheless, it is crucial to acknowledge the various challenges of utilizing wearable sensors as stress detectors. These challenges encompass concerns regarding the accuracy and reliability of the collected data and issues surrounding the privacy and security of the information. It is, therefore, essential to consider the practical implications of integrating wearable devices into stress management interventions [8].

iii) Research on stress prediction models: Reference to established models that utilize physiological data, such as heart rate variability (HRV) and sleep and breathing patterns (or even cortisol levels), for predicting stress levels. Continuous advancements in biotechnology are crucial for promoting and maintaining human health. These upgrades manifest in various aspects, including the integration of wearables, the implementation of data visualization, the support of artificial intelligence (AI) and machine learning (ML) in decision-making [9], and the incorporation of other state-of-the-art solutions. The review encompasses an assessment of the methodologies employed, the accuracy and reliability of predictions, and the limitations or challenges encountered in these models.

Respectively, stress responses encompass intricate physiological mechanisms designed to aid the body in addressing perceived threats or challenges. These responses are governed by diverse pathways and systems, including the autonomic nervous system (ANS) with its sympathetic and parasympathetic branches [10],[11], as well as the endocrine system, notably the hypothalamic-pituitary-adrenal (HPA) axis [12]. The sympathetic nervous system (SNS), often termed the “fight or flight” system, activates in response to perceived threats, triggering the release of neurotransmitters like norepinephrine and epinephrine [13]. This activation elicits physiological changes such as heightened HR, lung airway dilation, increased blood flow to muscles, and enhanced alertness. Conversely, the parasympathetic nervous system (PNS) orchestrates the body's “rest and digest” response [11], counterbalancing sympathetic activation by inducing relaxation responses like lowered HR, airway constriction, and improved digestion. Meanwhile, the HPA axis assumes a pivotal role in stress response regulation. Upon stress perception, the hypothalamus secretes corticotropin-releasing hormone (CRH), stimulating the pituitary gland to release adrenocorticotropic hormone (ACTH) [13]. ACTH then prompts the adrenal glands to release cortisol, the primary stress hormone. Cortisol regulates energy metabolism, inflammation suppression, and immune function modulation. Prolonged HPA axis activation and elevated cortisol levels can lead to adverse health effects, including compromised immune function and increased susceptibility to chronic diseases.

In terms of methodology and search strategies, the objective was to present a thorough examination of the subject matter within the confines of a concise review. The methodology encompassed targeted searches conducted across reputable academic databases, notably PubMed, Google Scholar, and pertinent conference proceedings. We employed a combination of specific keywords and phrases appropriate to the topic to identify relevant literature. The selection of references for Table 1 was based on the pertinence and significance of each publication to the themes under review, focusing on seminal works and recent contributions that significantly advanced understanding. Tables 3 and 5, detailing leading products/services and repositories, respectively, were compiled following a thorough review of publicly available resources. Factors such as impact and relevance to the overarching theme of the paper guided our selection process. This methodological rigor ensured a balanced and insightful exploration of the contemporary landscape of stress assessment and the utilization of wearable technology.

This review is organized as follows: Section 2 presents related work for stress monitoring, Section 3 provides details on the physiological indicators of stress, details on the wearable devices in stress monitoring, commercial products and services that utilize stress-detection software, discussion on existing stress prediction models, and reference to open repositories and databases of stress-related data. Finally, Section 4 presents a summary and suggestions for future work.

## 2. Related work

The identification of stress is a highly researched domain within the range of an individual's health, and numerous researchers have contributed to this field by presenting various approaches to measuring stress [14],[15]. Some have focused on “contactless” strategies, utilizing smartphone data (e.g., call/SMS logs, app usage, motion), voice processing, facial expression analysis, or keyboard typing behaviors. However, this review will focus on the use of wearable devices to measure physiological data for stress assessment. Notable works, like the one by Healey and Picard [16], demonstrated early success in detecting stress using physiological sensors. However, the environmental contexts in which stress detection is intended vary, ranging from constrained lab environments to unconstrained real-life scenarios. Some studies employ custom sensor suites, while others use commercially available sensors. The choice of sensors impacts factors like signal quality, reproducibility, and cost.

The literature review also highlights diverse ML approaches for stress detection, including rule-based techniques, factor graph models, ensemble learning, and deep learning. Various studies utilize different physiological features and classifiers, leading to a wide range of reported accuracies. Despite the considerable efforts, there remains an open space for further exploration and improvement of prediction accuracy. The complexity of developing reliable stress-monitoring devices is underscored by the need for more consensus on the optimal approach and the imperative for thorough statistical analyses. Previous works in physiological stress sensing have employed various wearable sensors, such as electrocardiography (ECG) sensors [17]–[20], electrodermal activity (EDA) sensors [21]–[24], inductive respiration (RIP) sensors [20],[22],[24],[25], blood volume pulse (BVP) finger clip sensor [26]–[28], and electromyography (EMG) sensors [29]–[33], and wearable devices such as Fitbit Sense, Empatica E4, or Shimmer GSR3+ [34]. These sensors have been utilized in diverse conditions, including lab-induced stress, constrained real-life activities (e.g., driving, call centers, sleeping), and free-living conditions [23].

In connection with the above, physiological measures like EDA, HR, and HRV are commonly used in studies related to well-being and affection [35]. Some studies propose smartwatch-based systems for discrete and cost-effective stress detection during daily activities. Kulkarni et al. [36] reviewed smart devices for health sensing from 2017 to 2022, covering diverse projects and methods for data collection and analysis, including exploring supplementary data for enhancing stress detection systems.

The role of classification algorithms and ML techniques in stress detection is pivotal, as outlined in **Table 3**. Numerous studies have utilized a spectrum of algorithms, including but not limited to Naive Bayes, K-Nearest Neighbors Algorithm (KNN), Decision Trees (DT), Support Vector Machines (SVM), Random Forests (RF), Multi-layer Perceptron, AdaBoost, and Logistic Regression. Additionally, Support Vector Machine (SVM), Bayesian Networks (BN), Artificial Neural Network (ANN), Fuzzy Logic, and other computer-aided diagnostic (CAD) tools have been applied for classification purposes [37]–[43].

In the existing literature, multiple studies have explored ensemble learning approaches in stress detection. For instance, Khullar et al. [44] introduced an ensemble model for stress detection utilizing physiological signals associated with anxiety. Issa [45] employed a two-step ensemble methodology for detecting stress in automobile drivers. Likewise, Di Martino & Delmastro [46] utilized an ensemble model for predicting physiological stress. Lee et al. [47] proposed an ensemble model that integrates deep learning models, including gated recurrent units, convolutional neural networks (CNN), and recurrent neural networks. Notably, the selection of sensors, data types, and classification algorithms varies among these studies, resulting in disparate classification accuracies.

Exploring stress identification through wearable devices and physiological data has witnessed significant advancements, with researchers employing diverse methodologies and technologies. Incorporating diverse physiological features and classifiers has led to a spectrum of reported accuracies. However, despite considerable efforts, there remains an open space for further exploration and enhancement of prediction accuracy. The significance of classification algorithms in stress detection cannot be overstated, with studies employing a spectrum of algorithms (**Table 3**). Ensemble learning approaches have been particularly prevalent, as evidenced by studies introducing models designed for stress detection in various contexts [22],[48].

The landscape of stress identification through wearable devices and physiological data is characterized by its complexity, diversity, and ongoing exploration. The integration of advanced technologies, ML techniques, and diverse physiological measures holds promise for the development of more accurate and reliable stress-monitoring devices in the future. However, the interdisciplinary nature of this field necessitates continued collaboration and research to address existing challenges and unlock the full potential of stress detection in diverse real-life scenarios [9],[34],[36],[40].

## 3. Physiological indicators of stress

### 3.1. Physiological indicators of stress

A fundamental consideration in the assessment of stress involves the examination of its diverse components. Initially, stress specific to testing situations was perceived as a unified and one-dimensional concept by Mandler and Sarason [49]. Liebert and Morris [50] later introduced a distinction between “worry” (cognitive) and “emotionality” (affective) components, deviating from the initial dichotomous perspective on anxiety presented by Scherer [51]. Subsequent research embraced a multicomponent framework, acknowledging the interrelated nature of cognitive, affective, motivational, and physiological elements, departing from the earlier binary viewpoint on anxiety. This widely recognized classification highlights the theoretical differences among these components while emphasizing their interconnectedness. Although theoretically distinct, these emotional components, within the range of stress, demonstrate interrelated characteristics. These components encompass intrusive cognitive thoughts, affective expressions of nervousness, a motivational tendency to withdraw from the situation, and heightened physiological arousal, such as an elevated HR, increased perspiration, and physiological arousal [52]–[54].

Physiological responses to stress span cardiovascular changes like heightened HR and blood pressure, respiratory alterations such as rapid breathing and muscular tension, cognitive enhancements in alertness and focus, metabolic shifts like increased glucose release for energy, and immunological adjustments involving the suppression of non-essential immune functions. Comprehending these physiological pathways and responses is vital for effectively assessing and managing stress. Monitoring parameters like HRV, cortisol levels, and immune function can offer valuable insights into an individual's stress levels and overall well-being.

Stress responses involve a complex interplay of physiological pathways aimed at aiding the organism in dealing with perceived challenges. These mechanisms involve the ANS, which consists of the sympathetic and parasympathetic divisions, and the endocrine system, particularly the HPA axis [12],[55]. Upon encountering stressors, the SNS activates, leading to the release of neurotransmitters like epinephrine and norepinephrine. This activation induces various physiological changes, including increased HR, bronchodilation, enhanced blood flow to skeletal muscles, and heightened alertness. In contrast, the PNS initiates the body's “rest and digest” response, opposing sympathetic arousal by reducing HR and bronchoconstriction and promoting gastrointestinal activity. Concurrently, the HPA axis regulates stress responses by releasing CRH from the hypothalamus and stimulating the anterior pituitary to release ACTH, which, in turn, triggers the adrenal glands to secrete cortisol, the primary stress hormone. Cortisol modulates various physiological processes, including energy metabolism, inflammation regulation, and immune function [13]. Prolonged HPA axis activation and elevated cortisol levels can lead to adverse health consequences, such as compromised immune function and increased susceptibility to chronic diseases. Physiological responses to stress encompass changes in cardiovascular function, respiratory patterns, muscle tension, cognitive function, metabolic adjustments involving increased glucose release, and alterations in immune resource allocation, prioritizing immediate threats. Additionally, stress can influence metabolic biomarkers, antioxidants, glucose levels, hemoglobin, C-reactive protein (CRP), cytokines (including pro-inflammatory and anti-inflammatory cytokines), and tumor necrosis factor (TNF). Furthermore, research indicates that stress can be assessed through various biomarkers like hair cortisol, salivary cortisol, and urinary cortisol levels, providing insights into the physiological impacts of stress on the body [54]–[58].

In the last two decades, there have been considerable advancements in stress assessment, coinciding with the development of physiological and biochemical sensing technologies. The sensors are a solid foundation for connected health solutions and proactive care in addressing various conditions related to or induced by stress [59],[60]. Stress is characterized as a disturbance in an individual's homeostatic balance, prompting the body to initiate what is known as the stress response [61]. Stress can be acute, an immediate reaction to a stressor, or chronic, a prolonged state resulting from constant stress stimuli [62]. Prolonged exposure to chronic stress may push the body to a point where it can no longer achieve a balanced state, rendering the individual unable to manage stressors effectively. The activation of the stress response induces various physiological changes driven by the stimulation of the SNS and the inhibition of the parasympathetic system. While the stress response can manifest differently, it typically involves the release of stress hormones that heighten the body's alertness. Consequently, there is an elevation in HR, blood supply to the muscles, respiratory rate, skin temperature (ST) (attributed to increased blood circulation), and cognitive activity, among other responses. Quantitative assessment or stress monitoring often involves analyzing stress-specific hormonal responses and other biomarkers affected by the stress response [61],[63].

Stress evaluation can be conducted through subjective means, employing structured questionnaires and self-reporting forms, which aligns with standard clinical practice. Alternatively, objective assessment measures various bodily responses to stress [64]. Standard tools in clinical stress evaluation include self-reported questionnaires, exemplified by Cohen's Perceived Stress Scale (PSS) [4], and self-reported visual scales, such as the Visual Analogue Scale for Stress (VASS) [65]. Biomedical researchers utilize biochemical markers like cortisol and α-amylase to detect stress, often inducing a stress state in subjects through the Trier Social Stress Test (TSST) [66]–[70].

Extensive literature exists on the monitoring of stress through the physiological or biochemical responses of the human body. However, a consensus remains elusive regarding the sensitivity and specificity of these physiological and biochemical indicators for identifying stress. The variability in sensitivity and specificity can be attributed to factors such as the responsiveness of the stress reaction, sensor sensitivity, the nature of stimulants, sample size in the study, experimental design, and other variables [41]. The literature review emphasizes the diverse range of wearable sensors that have been applied in various conditions, from controlled laboratory-induced stress to real-world activities and free-living scenarios. The selection of sensors underscores the necessity for adaptability to different stress-inducing situations. While there has been a notable surge in interest in physiological indicators over the past decade to complement traditional self-reports of emotions [71], applying these measures remains labor-intensive and costly, and interpreting findings within the context of existing educational research and theories remains challenging [10]. Until now, only a restricted number of studies have combined electrodermal measurements with self-reported stress assessments. Integrating electrodermal measures with self-report measures of anxiety is limited, with physiological measures commonly integrated into studies focused on well-being and affection. On the other side, smartwatch-based systems offer unobtrusive and cost-effective stress detection during daily activities, with devices like Empatica E4, Shimmer3 GSR+, or Movisense EdaMove providing a continuous assessment of physiological indicators, opening avenues for exploring the relationship among control, anxiety components, and daily performance.

Biochemical stress indicators offer better detection and monitoring capabilities, but a drawback is that many of these indicators require invasive measurement methods. Some examples include cortisol, which typically requires the collection of saliva, blood, or urine samples for analysis; catecholamines such as epinephrine and norepinephrine, which are usually measured through blood samples; and certain inflammatory markers like interleukin-6 (IL-6), which also require blood samples for assessment [54],[58],[72]. In cases where noninvasive measurement is feasible, extracting the necessary hormones from collected samples takes time, making real-time stress monitoring devices impractical. There is potential in combining biochemical indicators, such as cortisol level measurement, with physiological indicators like HR, respiratory rate, and activity monitoring to create more resilient and precise stress-monitoring devices. **Table 2** references biophysiological* and biochemical** stress parameters (sorted year-wise).

**Table 1.** Biophysiological* and biochemical** parameters of stress.

**Ref**	**Year**	**Signals**	**Stressors**
[68]	2010	ECG, photoplethysmography (PPG), EDA, and ST [*BPH]	Public speaking, mathematics, mental, social, and physical challenges
[63]	2010	Salivary alpha-amylase, plasma catecholamines, blood pressure, and HR [**BC]	College academic final exam
[55]	2010	EEG, EDA PPG, and respiratory [*BPH]	International Affective Picture System (IAPS)
[94]	2011	ECG, respiratory, EDA, and EMG [*BPH]	Perceived Stress Scale (PSS) questionnaire
[95]	2012	Hair cortisol [**BC]	Daily life stress (3 months)
[96]	2012	ECG, EDA, and accelerometer (ACC) [*BPH]	Stroop color test and mental arithmetic problems based on the Montreal Imaging Stress Task (MIST)
[97]	2013	Sweat and saliva samples [**BC]	Intense exercise
[98]	2013	ECG, EMG, galvanic skin response (GSR), and ST (only concern on ECG and HRV) [*BPH]	Stroop word–color test
[99]	2014	ECG, respiratory, body temp, GSR [*BPH]	Hajj pilgrimage (mandatory annual pilgrimage for all Muslims)
[69]	2015	EDA and PPG [*BPH]	Trier Scope Stress Test (TSST)
[97]	2015	ECG and thoracic electrical bioimpedance (TEB) measurements [*BPH]	Films game based on the addition
[100]	2016	Steroid hormones in hair [**BC]	Perceived Stress Questionnaire (PSQ)
[41]	2016	EDA, PPG, and sociometric badge for recording [*BPH]	STAI (State-Trait Anxiety Inventory) and TSST
[34]	2017	ECG, EDA, and respiratory [*BPH]	Real driving environment
[101]	2017	EDA, ST, accelerometer (ACC), and PPG [*BPH]	Randomly generated equations (solved verbally)
[102]	2017	ECG and respiratory [*BPH]	Montreal Imaging Stress Task (MIST)
[103]	2017	PPG and inertial motion and driver behavior [*BPH]	Euro truck driving simulator
[56]	2018	Biochemical (salivary cortisol) and physiological domains (HRV measures) [**BC]	Academic final examination, Psychological stress response inventory
[104]	2018	ECG [*BPH]	Daily life stress
[105]	2018	PPG, ACC, and EDA [*BPH]	City car driving simulator
[106]	2018	EDA only ECG, EMG, and respiratory [*BPH]	Driving on the highway in the city
[107]	2018	Arginine, phenylalanine, acylcarnitines, sphingomyelin [**BC]	Night shifts
[70]	2019	ST, HR pulse wave EDA, ECG, PPG, copeptin, prolactin (blood), cortisol, and alpha-amylase (saliva) [**BC]	Trier Social Stress Test (TSST)
[108]	2019	PPG and endocrine (salivary) cortisol [**BC]	Childhood Trauma Questionnaire (CTQ)
[108]	2019	Oxy-hemoglobin (oxy-Hb) [**BC]	Oxy-hemoglobin (oxy-Hb)
[108]	2019	PPG, EDA, GSR, and ACC [*BPH]	Summer camp (training, the contest, and free day)
[109]	2019	EDA [*BPH]	Driving on the highway in the city
[110]	2020	GSR [*BPH]	Predefined PYSIONET dataset and driving on the highway in the city
[111]	2021	Plasma cortisol [**BC]	Daily life stress
[112]	2021	Copeptin, neurophysin II, vasopressin [**BC]	Daily life stress
[113]	2021	Cortisol, adrenaline, alpha-amylase, copeptin, and prolactin [**BC]	Positive and Negative Affect Schedule (PANAS), State-Trait Anxiety Inventory (STAI), Self-Assessment Manikins questionnaire (SAM), Short Stress State Questionnaire (SSSQ)
[114]	2021	BVP, ST (TEMP), EMG, photoplethysmogram (PPG), and EDA [*BPH]	Montreal Imaging Stress Task (MIST)
[111]	2022	Salivary alpha-amylase from saliva sample [**BC]	Various stressors related to school engagement
[86]	2022	Salivary cortisol was analyzed using the IDS-iSYS Multi-Discipline Automated System (Immunodiagnostic Systems Limited) [**BC]	Triggered by stressors in individuals with regular, non-clinical functioning
[111]	2022	Oxidative stress (urine analyzed using an immunoassay kit OxiSelect™ 8-iso-Prostaglandin F2a ELISA Kit from Cell Biolabs, Inc) [**BC]	Various stressors related to school engagement and daily activities
[57]	2023	Salivary cortisol [analyzed cortisol awakening response (CAR) and the diurnal baseline cortisol (DBC)] [**BC]	Daily activities

### 3.2. Wearable devices in stress monitoring

Implementing an automated stress-monitoring system benefits the self-management of mental and, consequently, physical well-being in a wide spectrum of individuals navigating the stress-laden environments of modern society. The intricacies that render the monitoring of stress a challenging and research-worthy endeavor include: i) subjectivity of stress: stress is highly subjective, with stimuli triggering the stress process varying between individuals; ii) defining ground truth: establishing the ground truth for stress detection proves challenging due to the subjective and continuous nature of the stress process, making it difficult to delineate the onset, duration, and intensity of a stress event; iii) indirect monitoring of stress: the stress response encompasses physiological, behavioral, and affective components. While certain aspects of the physiological response can be directly monitored using wearable devices (e.g., increased HR, sweating rate), there are no direct methods for monitoring the behavioral and affective components.

Recent technological advancements have introduced wearable biosensors (e.g., ECG sensors, sweating-rate sensors [73], respiration-rate body sensors [74]) into daily life. The proliferation of wearables with bio-signal acquisition capabilities presents significant opportunities for advanced machine learning–enabled health monitoring and intervention applications. While literature demonstrates the feasibility of objectively detecting stress through biological signals, existing frameworks are often designed for controlled settings. Stress detection in everyday scenarios introduces inherent challenges such as real-time data collection and analysis, lower signal quality due to motion and noise artifacts (MNAs), and difficulties in collecting self-reports owing to limited user adherence [75]. Moreover, the personalization of stress monitoring in everyday settings poses additional challenges. User-specific features may emerge based on individual characteristics, behavioral patterns, physiology, context, and sensor setup/configuration, resulting in more variability than in controlled settings. These differences could degrade the performance of general classifiers in everyday scenarios.

Implementing an automated stress-monitoring system holds great promise for enhancing the self-management of mental and physical well-being in the complex landscapes of contemporary society. The challenges associated with stress monitoring have been acknowledged, including the subjective nature of stress, difficulty defining ground truth, and the indirect nature of monitoring certain components. Nevertheless, recent technological strides in wearable biosensors, encompassing GPRS body control sensors, Bluetooth trackers, and smart clothes sensors, have opened avenues for leveraging advanced ML in health monitoring and interventions. **Table 2** demonstrates the placement of different wearable biosensing devices used for stress monitoring.

**Table 2.** Summary of possible sites for wearable sensing technologies placement.

	**Biosensing devices**	
	GPRS body control	
	Smart glasses	
	Smart watch	
	Smart bracelets/wristbands	
	Smart ring	
	Bluetooth key tracker	
	Smart belt	
	Smart clothes (shirts, pants, shoes, socks)	

### 3.3. Commercial products and services for stress detection

Considering the heightened acknowledgment of health issues associated with stress and the advancements in wearable biosensor technologies, a spectrum of commercial products and services has arisen, integrating stress-sensing software into their functions.

Indicatively, they are divided into the following categories: i) smart wearables with embedded stress monitoring, ii) biofeedback devices, iii) stress-responsive mobile applications, iv) emotion recognition software, v) stress detection and monitoring platforms, etc.

Smart wearable devices, ranging from smartwatches to fitness trackers, employ sensors such as ECG sensors, sweating-rate sensors, and respiration-rate body sensors to capture physiological indicators associated with stress. These wearables conduct real-time analysis of the gathered data using advanced ML algorithms, providing users with immediate insights into their stress levels. In parallel, biofeedback devices, such as HRV monitors and galvanic skin response sensors, contribute additional layers of physiological data, enabling a more comprehensive understanding of the user's stress profile.

Mobile applications have been devised to complement stress-monitoring endeavors with wearable devices. These applications connect with wearables, collecting and consolidating data for a comprehensive stress analysis. Users can receive personalized stress reports, trend analyses, and actionable recommendations on managing stress. ML integration enables continuous accuracy improvement by adapting to individual stress response patterns over time.

Accordingly, emotion recognition software, wearable devices, and mobile applications gain enhanced capabilities. Emotion-recognition algorithms can analyze facial expressions, voice tone, and speech patterns to discern emotional states. Integrating this software with wearables and apps augments the understanding of stress by incorporating emotional context. This synergy enables stress-detection platforms to offer more nuanced and personalized stress management guidance, including tailored coping strategies and interventions aligned with the user's emotional and physiological state. As technology evolves, the convergence of biofeedback and emotion recognition further refines the precision and effectiveness of stress management on an individualized level. **Table 3** illustrates a sample of leading technological products and services employing stress-detection software.

**Table 3.** Leading technological products and services employing stress detection software.

**Type of device/Application**	**Commercial name**	**Parameters monitored**
Wearable device	Empatica E4	EDA, temperature, HRV, and motion measurement
Wearable device(s)	VivoSense	Wearable sensor technologies monitoring ECG, EMG, and EDA
Wearable device	Moodmetric Ring	EDA and stress level measurement
Wearable device	Fitbit	Stress tracking features, HRV, and stress management tools
Wearable device	Hexoskin Smart Shirt	ECG, HRV, and breathing rate monitoring
Wearable device	Oura Ring	HRV and sleep patterns monitoring
Wearable device	Garmin (Vivosmart)	HRV, various physiological metrics
Wearable device(s)	Amazfit Band Series	Stress level monitoring, HRV analysis
Wearable device	WHOOP Strap	HRV, stress, and recovery optimization
Wearable/biofeedback device(s)	Biostrap	HRV and sleep pattern monitoring, activity level tracking
Biofeedback device/app	Elite HRV	HRV monitoring
Biofeedback device	Wild Divine's IomP	EDA measurement, biofeedback games
Biofeedback device(s)/app	Shimmer3 GSR+ (Galvanic Skin Response)	Changes in skin conductance measurement, ST
Biofeedback device	HeartMath Inner Balance	HRV measurement, stress insights
Biofeedback device	EmWave by HeartMath	EmWave devices for HRV biofeedback
Biofeedback device	Spire Health Tag	Respiratory patterns tracking,
Biofeedback device/app	Muse	brain activity (EEG) measurement, HRV
Biofeedback Device/app/platform	Welltory	Biofeedback and HRV analyzation
Mobile app	Biospectal OptiBP	Monitoring blood pressure via smartphone's camera
Emotion recognition software/platform	Affectiva	Emotion recognition software, facial expression analysis, muscle tension
Emotion recognition software	Microsoft Azure Face API	Facial expression analysis, emotion detection, muscle tension
Emotion recognition software	Beyond Verbal	Vocal emotion analysis, speech patterns, emotion detection, muscle tension
Emotion recognition software	Noldus FaceReader	Facial expression analysis, emotion recognition, HRV
Emotion recognition software/app	iMotions	Facial expression analysis, a platform for biometric research
Platform	BioBeats	Combination of wearable devices and ML algorithms for stress monitoring, insights based on physiological data
Platform/App/Biofeedback Device	Unyte iom2	Stress detection and monitoring, HRV, ST
Platform/Wearable Devices	Neumitra	Physiological signals measurement, HRV, stress detection, and real-time feedback

While the feasibility of objectively detecting stress through biological signals has been demonstrated, the transition from controlled settings to everyday scenarios introduces inherent complexities. Real-time data collection and analysis, mitigating motion and noise artifacts, and overcoming challenges in obtaining self-reports due to limited user adherence pose significant hurdles. Furthermore, the personalization of stress monitoring in daily life adds another layer of difficulty, with user-specific features stemming from individual characteristics, behavioral patterns, physiology, context, and sensor configurations, contributing to heightened variability.

Despite the challenges mentioned above, the proliferation of wearable biosensing devices presents valuable opportunities for advancing stress-monitoring technologies. As illustrated in **Table 2**, the placement of various devices showcases the diversity in approaches. It underscores the need for continued research and innovation in tailoring stress-detection frameworks to meet the demands of real-world, everyday scenarios. As technology undergoes further advancements and corresponding products and services enter the commercial sphere, as indicated in **Table 3**, addressing these challenges will be crucial in realizing the full potential of automated stress monitoring for improving overall well-being in diverse populations.

### 3.4. Stress prediction models

In the past, researchers have employed various devices and sensors to record physiological signals. While there is an overlap in the physiological signals measured across different studies, more needs to be explored into whether machine-learning models trained on data from one device can effectively generalize to the same physiological signal collected from a different device type. Research efforts have established a standard stress-detection framework involving signal cleaning, data normalization, segmentation, feature computation, classifier training, and performance assessment.

As previous research has established a groundwork for stress detection, extensive research has concentrated on optimizing these steps by experimenting with different normalization techniques, cleaning methods, features, window sizes, and machine-learning models. Mishra et al. proposed a two-layered approach, considering stress in the preceding minute when making inferences about the current minute. This significantly enhances performance compared with standard single-model classification [76].

While stress-detection models based on physiological sensing demonstrate efficacy in controlled lab environments, their performance degrades when deployed in free-living conditions—a consistent trend across studies. A primary reason for this discrepancy is that the wearable sensors utilized in these studies measure the body's physiological response to stress rather than stress itself. Free-living conditions can induce physiological responses that confound stress-detection models. Prior efforts have addressed this by accounting for physical activity in free-living conditions, improving model performance. However, focusing solely on physical activity may prove insufficient. Manual methods for stress measurement, such as examinations by psychologists and psychiatrists, are available but have disadvantages like cost and time. Technology offers an alternative, providing cost-effective and timely results. ML has been employed in stress detection, with studies utilizing models like random forest and support vector machines trained on physiological features. A hybrid ML model, combining two models, has shown promise in generating more significant stress detection results in accuracy and efficiency.

Data from sensors undergo feature extraction, and ML or pattern recognition is employed to distinguish between stress and non-stress states (or baseline). ML algorithms fall into two primary categories. The first is supervised learning, where input and classification labels are provided to the model for prediction and classification. The second is unsupervised learning, where no labels are provided, and the model groups input data based on inherent patterns or similarities. Typically, sensor data is recorded on the device and transmitted to a computer or the cloud for processing and analysis. In certain scenarios, such as simulated driving, participants' wearable sensors are directly linked to a computer for real-time analysis during the experiment. As mentioned, various studies have utilized various algorithms, ML techniques, and other CAD tools (**Table 4**) for classification purposes [9],[30],[31],[35],[40].

**Table 4.** Summary of machine learning algorithms used in stress assessment.

**Algorithm**	**Information**
**Naive Bayes**	Naive Bayes is a probabilistic algorithm based on Bayes' theorem. It assumes independence between features, making it computationally efficient. It is widely used in classification tasks, particularly in natural language processing.
**Bayesian Networks (BN)**	Bayesian Networks represent probabilistic relationships among variables using a directed acyclic graph. They are employed for reasoning under uncertainty and are valuable in medical diagnosis, risk assessment, and decision-making systems.
**K-Nearest Neighbors Algorithm (KNN)**	KNN is a simple and effective algorithm used for classification and regression tasks. It classifies data points based on the majority class of their nearest neighbors.
**Decision Trees (DT)**	Decision Trees are tree-like models that make decisions based on features. They are widely used for classification and regression. Decision trees are interpretable but prone to overfitting, which techniques like pruning can address.
**Support Vector Machines (SVM)**	SVM is a powerful algorithm for classification and regression tasks. It identifies a hyperplane that best separates data into classes. SVM is effective in high-dimensional spaces and is commonly used in image classification and bioinformatics.
**Random Forests (RF)**	Random Forests are an ensemble-learning method based on multiple decision trees. They enhance accuracy and mitigate overfitting by aggregating the predictions of individual trees. Random Forests are widely used for diverse machine-learning tasks.
**Multi-Layer Perceptron**	Multi-Layer Perceptron is an artificial neural network with multiple layers, including input, hidden, and output layers. It excels in learning complex patterns and is widely used in deep learning applications.
**AdaBoost**	AdaBoost is an ensemble-learning algorithm that combines weak learners to create a strong classifier. It assigns more weight to misclassified data points, emphasizing their importance in subsequent iterations.
**Logistic Regression**	Logistic Regression is a statistical method used for binary classification. Despite its name, it is a linear model widely used when the relationship between the dependent variable and predictors is logistic.
**Convolutional Neural Network (CNN)**	CNN is a deep learning algorithm for processing structured grid data like images. It utilizes convolutional layers to learn hierarchical features automatically. CNNs are widely employed in image recognition tasks.
**Artificial Neural Network (ANN)**	ANN is a computational model inspired by the human brain. Comprising interconnected nodes (neurons), it can learn complex relationships in data. ANNs are used in various fields, including finance, healthcare, and image processing.
**Fuzzy Logic**	Fuzzy Logic deals with uncertainty and imprecision in data by allowing partial membership to a set. It is employed in control systems, decision-making, and AI applications where exact reasoning is challenging.
**Computer-Aided Diagnostic (CAD) Tools**	CAD tools utilize various algorithms, including ML and statistical methods, to assist in medical diagnoses. They analyze medical data such as images, helping healthcare professionals make more informed decisions.

### 3.5. Open repositories of stress-related data

The developing discipline of stress detection via wearable technology has emphasized the significance of accessible and diverse datasets for progressing respective research. Acknowledging the important role of data in formulating and validating stress-detection models, various initiatives have been introduced to establish open repositories dedicated to stress-related physiological signals. These repositories support researchers by facilitating the collaborative exploration of stress-related datasets, contributing to the reproducibility and comparability of studies, and promoting a more robust understanding of stress detection across various contexts and populations.

A noteworthy initiative in this domain involves the aggregation of datasets encompassing physiological signals derived from a spectrum of wearable devices. These datasets systematically incorporate a range of stress-inducing scenarios, including controlled lab environments and free-living conditions. Including diverse stressors and real-world contexts aims to address the observed performance degradation of stress-detection models when transitioning from controlled settings to everyday life.

These open repositories feature raw physiological data and provide comprehensive annotations, including ground-truth labels for stress and non-stress states. The availability of labeled data is paramount for the training and evaluation of machine-learning models. Furthermore, some repositories incorporate metadata detailing the type of wearable sensors used, cleaning and normalization techniques applied, and other pertinent information that enhances the interpretability and generalizability of the datasets.

**Table 5** includes references to various open repositories that provide stress-related data for formulating and validating stress-detection models.

**Table 5.** Open repositories of stress-related data.

**Ref**	**Repository/Database**	**Description**
[115]	WESAD (wearable stress and affect detection) (2018)	A publicly available dataset for wearable stress and affect detection research.
[116]	AMIGOS (dataset for affect, personality, and mood research on individuals and groups) (2021)	A dataset designed for emotion and stress recognition research, capturing physiological signals in response to multimedia stimuli.
[117]	MAHNOB-HCI (multimodal database for affective computing and human-computer interaction)	A multimodal dataset containing physiological signals for research in affective computing and human-computer interaction.
[118]	DREAMER: a database for emotion recognition through EEG and ECG signals from wireless low-cost off-the-shelf devices	A dataset designed for emotion recognition research, measuring EEG and ECG signals in response to audio and visual stimuli in film.
[119]	SWELL: the swell knowledge work dataset for stress and user modeling research	A dataset containing data to study stress and user modeling collected from participants while performing work activities.
[120]–[124]	SEED (stress recognition using EEG and EDA databases)	Focuses on stress recognition using electroencephalogram (EEG) and EDA signals
[125]	HEROES: a video-based human emotion recognition database	A database for motion identification (whole-body movements) and recognition.
[72]	AffectNet database	A database for facial expression, valence, and arousal estimation related to stress and emotion research.
[126]	DEAP (database for emotion analysis using physiological signals)	A database containing physiological and electroencephalogram (EEG) signals for emotion analysis research.
[3]	BioVid heat pain database	It investigates physiological responses to heat pain stimuli for pain and stress research.
[127]	PHYSIOLAB dataset	A dataset containing physiological signals for studying stress and emotion in realistic scenarios.
[128]	AMASS (affect, motivation, and ambiance stress study)	A dataset for studying the relationship between stress and emotions in realistic environments.
[129]	CREMA-D (crowd-sourced emotional multimodal actors dataset)	A dataset for emotion and affective computing research, including physiological signals and facial expressions.
[130]	Multi-modal stress dataset (MMSD) for real-time, continuous stress detection	MMSD is a multimodal acute stress dataset that includes physiological signals (ECG, PPG, EDA, and EMG) for stress detection.
[131]	The University of Waterloo Stress Dataset (UWS)	UWS dataset contains physiological data, including HRV, EDA, and ECG, for stress identification in daily life conditions.
[132]	OpenFace Project	The OpenFace project provides tools for facial behavior analysis, which can be used in stress and emotion research.

In summary, the accessibility of open repositories significantly facilitates the advancement of research by fostering collaboration and sharing datasets. Particularly significant is the available platform IEEE DataPort [77] (hosting various datasets, including those related to physiological signals and stress) and free repositories, such as the Open Science Framework (OSF) [78], a comprehensive platform supporting the entire research lifecycle, where researchers commonly disseminate their datasets. Zenodo [79], developed under the European OpenAIRE program, stands as a general-purpose open-access repository accepting datasets from diverse scientific fields. Figshare [80] offers researchers a platform to publicly share various research outputs, including datasets, while Data.gov [81] provides a comprehensive resource spanning diverse domains, offering access to numerous datasets that may benefit researchers exploring stress-related phenomena. Additionally, the NIH Data Sharing Repositories [82] curated by the National Institutes of Health serve as a valuable reference, encompassing various biomedical and health-related research areas. These open repositories contribute to the open science ethos and enhance accessibility and collaboration within the broader research community.

## 4. Conclusions

In summary, this scientific review explores the intricate domain of stress assessment, particularly on incorporating wearable technology for the objective evaluation and real-time prediction of stress levels. The pervasive nature of stress in contemporary society accentuates the imperative for proactive interventions, and the emergence of wearable devices represents the necessity for addressing this imperative. The investigation into physiological stress indicators underscores the importance of metrics that can be seamlessly captured through wearable devices. Particularly noteworthy is the continuous monitoring of parameters such as HR and breathing patterns, providing real-time data and valuable insights into individuals' health. The correlations between these physiological markers and stress levels are examined, focusing on comprehending the consistency of these associations across diverse populations.

With their capability for real-time data acquisition, wearable devices assume a pivotal role in monitoring stress. The review acknowledges their effectiveness in providing individuals with immediate feedback on health indicators, facilitating the timely detection of patterns indicative of stress or anxiety. Nonetheless, the review underscores the challenges associated with these devices, encompassing data accuracy, reliability, privacy, and security considerations. Furthermore, emphasis is placed on the imperative to formulate efficacious interventions based on the collected data, thereby underscoring the practical ramifications associated with integrating wearables into stress management strategies. The findings of Burtscher et al. emphasize the potential for wearables to extend beyond general stress management, particularly in addressing non-motor symptoms in Parkinson's disease [83]. This fact highlights a promising avenue for future research and application, showcasing the broader impact of wearable systems on managing various stress-related symptoms across different medical conditions.

The integration of wearable technology within clinical environments presents promising avenues for personalized medicine. These devices can facilitate early diagnosis, continuous remote monitoring, and customized interventions across various medical conditions [85]–[87],[133]. Ongoing advancements in wearable technology, characterized by enhancements in sensor capabilities and algorithmic sophistication, can significantly augment healthcare professionals' capacity to evaluate and address stress-related symptoms among patients with diverse clinical profiles [86],[87].

As wearable technology emerges as a versatile tool for clinical practice, the deployment of sophisticated biosensors within these devices stands poised to enable the timely detection of cardiovascular events through the meticulous monitoring of physiological parameters such as heart rate variability and blood pressure [87],[88]. Likewise, integrating respiratory monitoring functionalities within wearables offers promising avenues for managing chronic respiratory ailments such as asthma and chronic obstructive pulmonary disease (COPD) [86],[89],[90]. Furthermore, the convergence of wearable glucose monitors with automated insulin delivery systems represents a notable stride in diabetes management, facilitating continuous monitoring of blood glucose levels and responsive insulin administration [91]. Furthermore, incorporating physiological sensors and machine learning algorithms into wearable devices implies significant strides in identifying and managing mental health disorders [5],[39],[92]. Additionally, wearable motion sensors hold considerable promise in rehabilitation and physical therapy, offering precise monitoring of movement patterns and real-time feedback to optimize therapeutic interventions [93]. These examples emphasize the transformative potential of wearable technology in healthcare, underscoring its capacity to afford personalized monitoring and intervention across various medical conditions.

The investigation into established stress prediction models reveals the continuous progression of biotechnology, coupled with its integration with state-of-the-art solutions, including AI and ML. The evaluation encompasses the methodologies utilized, the precision and dependability of predictions, and the encountered limitations or challenges. This comprehensive analysis contributes to a differentiated comprehension of the current landscape, establishing a foundation for future advancements in stress prediction models. Exploring stress prediction models reveals advancements in biotechnology, integrating AI and ML. The review assesses methodologies, precision, and limitations, contributing to a nuanced understanding of the current landscape and laying the foundation for future advancements. However, fundamental difficulties persist, including lacking a universally accepted definition of stress, physiological responses to stressors, and confounding factors like physical activity, that challenge stress-detection models. Reproducibility issues arise from custom hardware usage without testing applicability across devices, studies, populations, or demographics. Moreover, stress monitoring is challenging due to subjectivity, making it difficult to define the ground truth. Stress events' start, duration, and intensity are elusive, and the threefold stress response (physiological, behavioral, and affective) complicates direct monitoring. The difficulty in monitoring behavioral and affective responses highlights the need for innovative approaches.

The impact of stress on individuals and society is significant, and wearable devices hold great potential for stress management. However, it is crucial to approach wearable technology integration carefully, considering its capabilities and limitations. This review highlights this field's achievements while emphasizing the need for further investigation. Collaborative efforts among researchers, healthcare professionals, and technology developers are necessary to fully harness wearables' transformative potential in stress management. Despite technical challenges, wearables have significant potential to mitigate the adverse effects of stress and contribute to improved individual and public health outcomes.

## Use of AI tools declaration

The authors declare they have not used Artificial Intelligence (AI) tools in the creation of this article.
